# Recent trends in breast cancer incidence in US white women by county-level urban/rural and poverty status

**DOI:** 10.1186/1741-7015-7-31

**Published:** 2009-06-26

**Authors:** Amelia K Hausauer, Theresa HM Keegan, Ellen T Chang, Sally L Glaser, Holly Howe, Christina A Clarke

**Affiliations:** 1 Northern California Cancer Center, Fremont, CA, USA; 2 University of California San Francisco School of Medicine, University of California San Francisco, San Francisco, CA, USA; 3 Division of Epidemiology, Department of Health Research and Policy, Stanford School of Medicine, Stanford University, Stanford, CA, USA; 4 North American Association of Central Cancer Registries, Springfield, IL, USA

## Abstract

**Background:**

Unprecedented declines in invasive breast cancer rates occurred in the United States between 2001 and 2004, particularly for estrogen receptor-positive tumors among non-Hispanic white women over 50 years. To understand the broader public health import of these reductions among previously unstudied populations, we utilized the largest available US cancer registry resource to describe age-adjusted invasive and *in situ *breast cancer incidence trends for non-Hispanic white women aged 50 to 74 years overall and by county-level rural/urban and poverty status.

**Methods:**

We obtained invasive and *in situ *breast cancer incidence data for the years 1997 to 2004 from 29 population-based cancer registries participating in the North American Association of Central Cancer Registries resource. Annual age-adjusted rates were examined overall and by rural/urban and poverty of patients' counties of residence at diagnosis. Joinpoint regression was used to assess trends by annual quarter of diagnosis.

**Results:**

Between 2001 and 2004, overall invasive breast cancer incidence fell 13.2%, with greater reductions among women living in urban (-13.8%) versus rural (-7.5%) and low- (-13.0%) or middle- (-13.8%) versus high- (-9.6%) poverty counties. Most incidence rates peaked around 1999 then declined after second quarter 2002, although in rural counties, rates decreased monotonically after 1999. Similar but more attenuated patterns were seen for *in situ *cancers.

**Conclusion:**

Breast cancer rates fell more substantially in urban and low-poverty, affluent counties than in rural or high-poverty counties. These patterns likely reflect a major influence of reductions in hormone therapy use after July 2002 but cannot exclude possible effects due to screening patterns, particularly among rural populations where hormone therapy use was probably less prevalent.

## Background

Between 2001 and 2004, incidence rates of invasive breast cancer declined more than 8% in the United States (US), with the greatest drops observed for estrogen receptor-positive (ER+) tumors among women aged 50 years and over [1-8]. Given that breast cancer incidence had increased continuously since at least the mid-1930s [9], there has been great scientific and public interest in explaining these declines, with the following main hypotheses set forth: (1) widespread discontinuation of and/or failure to initiate postmenopausal estrogen/progestin hormone therapy (HT) after the early termination of the Women's Health Initiative (WHI) HT trial in July 2002, a change which would lower incidence by decreasing the prevalence of a presumed occult tumor promoter [1,2,5,8,10-15]; (2) the saturation of mammographic screening programs between 1998 and 2000, as a consequence of reductions in the pool of previously unscreened women, which would also lower incidence [2,7,16,17]; (3) an acute drop in the number of women receiving mammograms after 2001, thereby decreasing detection of early stage tumors to again result in lower incidence [16-19]; and (4) a combination of (1) to (3). These hypotheses have been discussed in relation to incidence rates over the past three decades [2,7] and in the context of uniformly screened populations [6], but the degree to which each influenced recent trends still remains unclear.

Since the US does not utilize any comprehensive, linked health-tracking databases, distinguishing among these explanations in an attempt to better inform breast cancer prevention strategies necessarily requires detailed assessment of population-based data by patient demographic and tumor characteristics. Using the National Cancer Institute's Surveillance Epidemiology and End Results (SEER) resource, we and others [1,2] have found that the incidence reductions were most pronounced for hormone receptor-positive tumors among non-Hispanic white women over 50 years of age but were virtually absent among African-American and women under 50 years at diagnosis [5,20]. These data underscore the clear variation in post-WHI incidence trends according to racial/ethnic-, age-, and tumor characteristic-defined subgroups and generally support the argument that recent breast cancer declines stemmed largely from mass HT discontinuation/non-initiation after second quarter (Q2) 2002 [1,5-7,21]. If declines did in fact result largely from changes in HT, then it is likely that incidence rates also vary by rural/urban and poverty status, because HT users were more likely to have higher education and lower poverty status than non-HT users [22]. To our knowledge, no studies have yet described incidence changes in US regions outside the SEER program, which are important for understanding how rate changes may have affected populations other than those living in the disproportionately urban areas included in SEER [23]. To this end, we took advantage of the North American Association of Central Cancer Registries (NAACCR) database, the largest US resource allowing for description by annual quarter of diagnosis and area-based measures of urbanicity and poverty, to determine how recent incidence changes affected these communities. With these data, we described age-adjusted invasive and *in situ *breast cancer incidence trends overall and by county-level measures of rural/urban and poverty status.

## Methods

### Cancer incidence rates

We obtained all breast cancer incidence data from NAACCR's research file (Cancer in North America [CINA] Deluxe for NHIAv2 Origin) for 475,523 cases of invasive and 111,885 cases of *in situ *breast cancer (*International Classification of Disease for Oncology, 3*^*rd*^*Edition *[ICD-O-3], sites 50.0–50.9) diagnosed between 1997 and 2004 and reported to 29 population-based cancer registries. These registries (Alaska; Atlanta, GA; California; Colorado; Connecticut; Detroit, MI; District of Columbia; Florida; Hawaii; Idaho; Illinois; Iowa; Kentucky; Louisiana; Maine; Massachusetts; Montana; Nebraska; Nevada; New Jersey; New Mexico; New York; Ohio; Oregon; Pennsylvania; Rhode Island; South Carolina; Texas; Washington) were selected, because they (1) met rigorous NAACCR criteria for completeness and quality [24], (2) submitted data continuously during the period 1997 to 2004, and (3) actively consented to participate in this analysis. Their combined catchment areas represented approximately 69% of the total US population during the observation period. Reported rates were highest in Washington DC, California, and Hawaii and lowest in Kentucky, Louisiana, and Ohio. Population estimates, based on US Census data, were obtained for these regions from the National Cancer Institute.

Because post-WHI breast cancer incidence trends have been shown to vary by patient race/ethnicity, age, invasiveness, and hormone receptor subtype [1-3,5-7,20] we limited analyses to non-Hispanic white (hereafter referred to as white) women aged 50 to 74 years. Women in this racial/ethnic age group were selected since they experienced larger incidence declines after 2002 [1,2,7] than women of other races/ethnicities and ages and are relatively homogenous with respect to menopausal status, HT use, regularity of mammographic screening, and other breast cancer risk factors (for example, familial risk) [25]. We examined incidence rates separately for invasive and *in situ *breast tumors but could not assess trends by tumor hormone receptor or HER2/neu status, as this information was not collected by most participating registries.

Rates were then stratified by two characteristics of patients' counties of residence at the time of diagnosis: rural/urban status and percentage of the population living under the federal poverty level (FPL). Rural/urban codes were assigned according to the 2003 US Department of Agriculture nine-point rural/urban codification scheme, which distinguishes counties by population size, degree of urbanization, and adjacency to a metropolitan area [26]. We further categorized rural/urban status as: (1) urban (2003 rural/urban continuum codes 1, 2, 3: counties in metropolitan areas of 1 million population or more, counties in metro areas of 250,000 to 1 million population, counties in metro areas of fewer than 250,000 population); (2) suburban (2003 rural/urban continuum codes 4, 5, 6: urban population of 20,000 or more adjacent to a metropolitan area, urban population of 20,000 or more not adjacent to a metropolitan area, urban population of 2,500 to 19,999 adjacent to a metropolitan area); or (3) rural (2003 rural/urban continuum codes 7, 8, 9: urban population of 2,500 to 19,999 not adjacent to a metropolitan area, completely rural or less than 2,500 urban population adjacent to a metropolitan area, completely rural or less than 2,500 urban population not adjacent to a metropolitan area). Poverty was categorized into three levels: (1) low (<5.0% and 5.0 to 9.9% of population below FPL); (2) middle (10.0 to 19.9% of population below FPL); or (3) high (20.0+% of population below FPL). The percentage of the total county population below the FPL, obtained through linkage with the US Census Bureau [27], has been shown previously to correlate well with other measures of economic deprivation including educational attainment, unemployment rate, and occupational composition [28]. A total of 137 cases of invasive and 37 cases of *in situ *breast cancer with unknown or missing counties of residence at diagnosis were excluded from these assessments.

We used SEER*Stat version 6.3.6 (National Cancer Institute, Bethesda, MD, USA) to calculate annual and quarterly incidence rates per 100,000 person-years (unless otherwise noted) and corresponding 95% confidence intervals (CI). All rates were age-adjusted to the 2000 US standard and were compared statistically by calculating the difference between 2001 and 2004 rates, dividing by the standard error of the difference, and testing this number for a statistically significant difference from zero using the standard normal distribution [29]. Tests of statistical significance assumed a two-sided *P *value of 0.05. To further characterize incidence patterns, we assessed quarterly rate trends using Joinpoint, version 3.0 (National Cancer Institute, Bethesda, MD, USA), which identifies significant changes in overall trends by fitting a series of linear regression functions based on a Monte Carlo permutation method [30]. Quarterly, rather than annual, rates were subjected to Joinpoint analysis to provide more points for regression as well as to identify more precisely the timing of rate changes. All data were plotted on a semi-logarithmic scale to aid visual assessment of slope differences [31]. NAACCR Institutional Review Board approved this project, and the Scientific Editorial Board approved the final manuscript.

## Results

Table 1 shows characteristics of the study patients and their tumors. Higher proportions of women diagnosed with breast cancer resided in urban than rural and low- or middle- than high-poverty counties, irrespective of invasive status.

**Table 1:** Characteristics of invasive and *in situ *breast cancer cases among non-Hispanic white women aged 50 to 74 years, 1997 to 2004 (NAACCR, CINA Deluxe).

	Invasive (*n *= 651,395)		*In situ* (*n *= 145,216)	
Characteristic	*n*	%	*n*	%

Rural/urban status				

Urban	407,202	85.7	98,473	88.0

Suburban	52,304	11.0	10,341	9.2

Rural	15,891	3.3	3,035	2.7

County poverty level				

Low-poverty counties (<10% below FPL)	191,562	40.3	49,449	44.2

Middle-poverty counties (10 to19.9% below FPL)	256,910	54.0	56,716	50.7

High-poverty counties (20+% below FPL)	26,925	5.7	5,684	5.1

FPL = federal poverty level.

### Annual incidence rate changes

**Figure 1:**
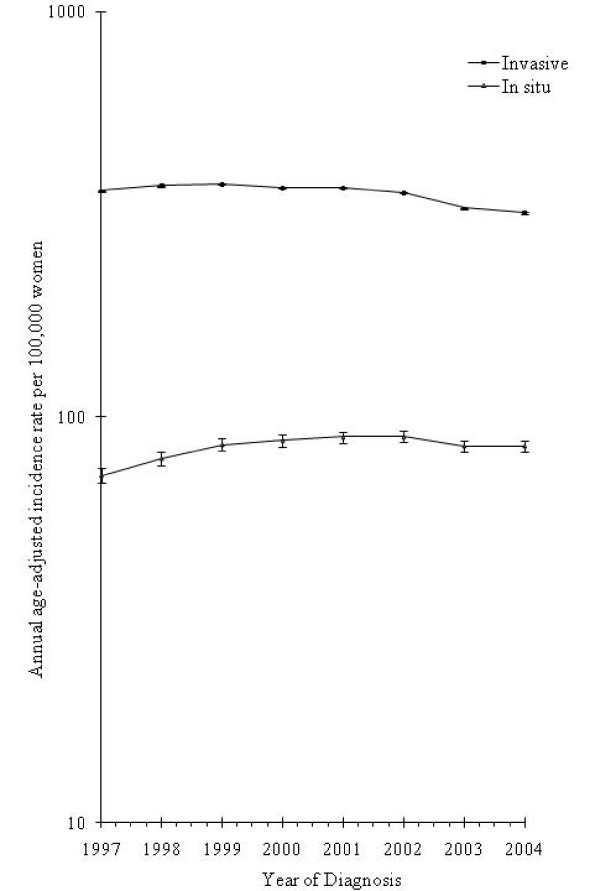
**Breast cancer incidence among non-Hispanic white women aged 50 to 74 years by invasive status and year**. **(a) **Trends for invasive breast cancer. **(b) **Trends for *in situ *breast cancer. All rates are age-adjusted to the 2000 US standard (NAACCR, CINA Deluxe).

**Figure 2:**
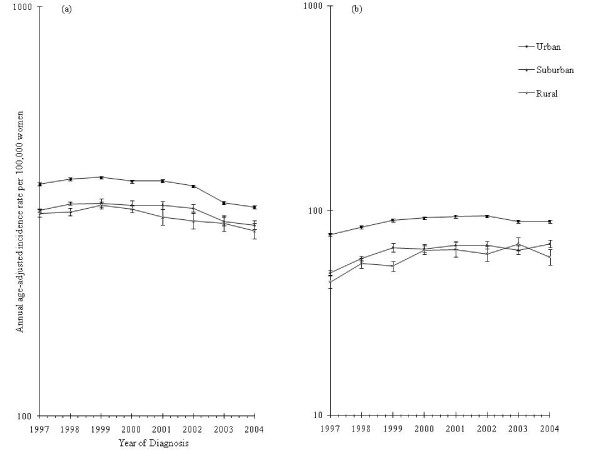
**Breast cancer incidence among non-Hispanic white women aged 50 to 74 years by county rural/urban status and year**. **(a) **Trends for invasive breast cancer. **(b) **Trends for *in situ *breast cancer. All rates are age-adjusted to the 2000 US standard (NAACCR, CINA Deluxe).

**Figure 3:**
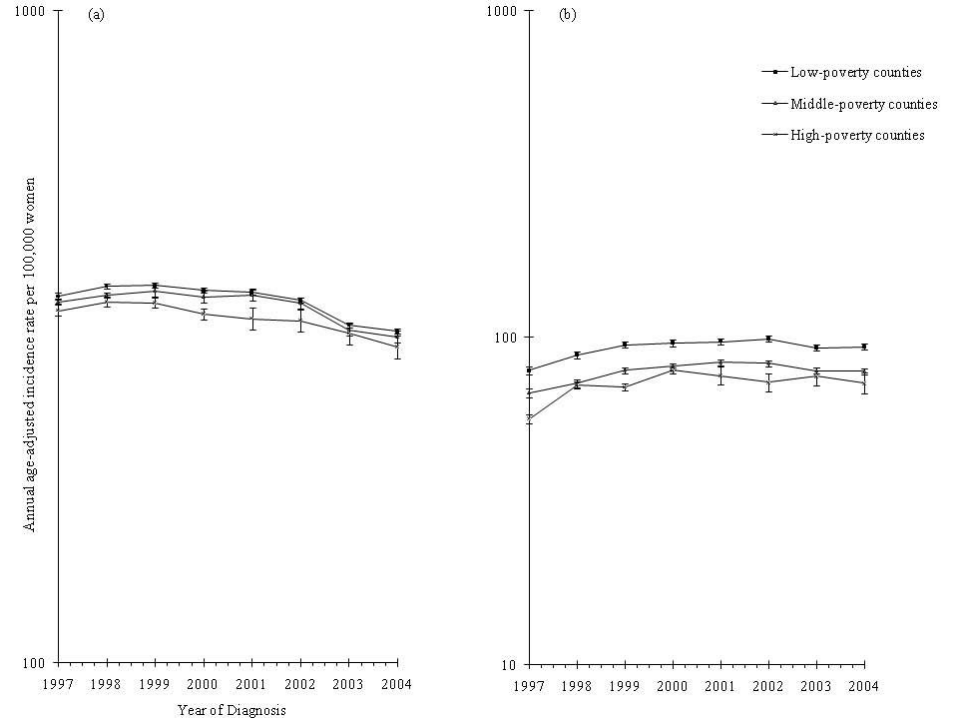
**Breast cancer incidence among non-Hispanic white women aged 50 to 74 years by county poverty level and year**. **(a) **Trends for invasive breast cancer. **(b) **Trends for *in situ *breast cancer. All rates are age-adjusted to the 2000 US standard (NAACCR, CINA Deluxe).

Figure 1 documents incidence trends according to tumor invasive status. Overall patterns of invasive breast cancer were comparable among women living in both urban and suburban counties but differed for women in rural counties (Figure 2). Although well-differentiated in previous years, county poverty level-specific rates of invasive tumors converged after 2002 (Figure 3A).

### Overall changes between 2001 and 2004

To be comparable with prior reports [1,2,20] describing rate drops between the years 2001 and 2004, we calculated absolute and relative incidence changes over this same time period (Table 2). For invasive cancers, overall rates fell 13.2%. Moreover, while rural and high poverty counties consistently reported lower incidence rates, changes were greater for urban (13.8%) and low (13.0%) or middle (13.8%) poverty counties. Most reductions were statistically significant (*P *< 0.05). Incidence of *in situ *cancers also declined by 4.8%.

**Table 2:** Absolute and relative percent changes in invasive and *in situ *breast cancer rates among non-Hispanic white women aged 50 to 74 years by county rural/urban and poverty status for the years 2001 and 2004 (NAACCR, CINA Deluxe).

	2001 rate (95% CI)	2004 rate (95% CI)	Absolute change (95% CI)	Relative change	*P *value for difference
	Invasive				

Overall	366.7 (363.8 to 369.6)	318.4 (315.8 to 321.0)	-48.3 (-52.19 to -44.41)	-13.2%	<0.001

Rural/urban status					

Urban	375.1 (371.9 to 378.3)	323.5 (320.6 to 326.4)	-51.6 (-55.9 to -47.3)	-13.8%	<0.001

Suburban	327.9 (320.1 to 335.8)	292.9 (285.7 to 300.2)	-35 (-45.7 to -24.3)	-10.7%	<0.001

Rural	306.2 (292.9 to 320.1)	283.1 (270.5 to 295.1)	-23.1 (-41.6 to -4.5)	-7.5%	0.02

County poverty level					

Low-poverty counties (<10% below FPL)	370.4 (365.8 to 375.1)	322.4 (318.2 to 326.6)	-48 (-54.3 to -41.8)	-13.0%	<0.001

Middle-poverty counties (10 to 19.9% below FPL)	367.0 (363.1 to 371.0)	316.5 (312.9 to 320.1)	-50.5 (-55.8 to -45.2)	-13.8%	<0.001

High-poverty counties (20+% below FPL)	337.6 (326.2 to 349.2)	305.1 (294.5 to 316.1)	-32.5 (-48.2 to -16.8)	-9.6%	<0.001

	*In situ*				

Overall	89.0 (87.6 to 90.4)	84.7 (83.3, 86.1)	-4.3 (-6.2 to -2.4)	-4.8%	<0.001

Rural/urban status					

Urban	93.2 (91.6 to 94.8)	88.1 (86.5 to 89.6)	-5.1 (-7.3 to -2.9)	-5.5%	<0.001

Suburban	67.5 (64.0 to 71.2)	68.7 (65.2 to 72.3)	+1.2 (-3.8 to +6.2)	+1.7%	0.6

Rural	64.3 (58.2 to 70.8)	59.1 (53.4 to 65.2)	-5.2 (-13.8 to +3.4)	-8.1%	0.2

County poverty level					

Low-poverty counties (<10% below FPL)	97.4 (95.1 to 99.8)	93.9 (91.6 to 96.1)	-3.5 (-6.7 to -0.3)	-3.6%	0.03

Middle-poverty counties (10 to 19.9% below FPL)	84.0 (82.2 to 86.0)	78.9 (77.1 to 80.7)	-5.1 (-7.7 to -2.5)	-6.1%	<0.001

High-poverty counties (20+% below FPL)	76.5 (71.1 to 82.2)	72.6 (67.4 to 78.0)	-3.9 (-11.5 to +3.7)	-5.1%	0.3

CI = confidence interval; FPL = federal poverty level

### Joinpoint regression analysis

Joinpoint regression analysis by annual quarter of diagnosis allowed for more detailed investigation of recent breast cancer patterns (Table 3). Four trends were detected for all invasive tumors and among women living in urban, low-, and middle-poverty counties: an incidence increase, stabilization, sharp but statistically non-significant decrease, followed by a second stabilization. The most rapid declines in these groups occurred between Q2 2002 and Q1 2003, whereas more gradual decreases began between Q2 1998 and Q1 1999 among women living in rural and high-poverty counties.

**Table 3:** Results from Joinpoint regressions for invasive and *in situ *breast cancer among non-Hispanic white women aged 50 to 74 years by county rural/urban and poverty status, 1997 to 2004 (NAACCR, CINA Deluxe).

	Trend time period	Quarterly % change	95% CI
Invasive			

Overall	Q1 1997 to Q2 1998	+1.2	+0.04 to +2.4

	Q2 1998 to Q2 2002	-0.2	-0.4 to +0.1

	Q2 2002 to Q1 2003	-3.6	-8.5 to +1.6

	Q1 2003 to Q4 2004	-0.4	-1.1 to +0.4

Rural/urban status			

Urban	Q1 1997 to Q2 1998	+1.2	+0.04 to +2.4

	Q2 1998 to Q2 2002	-0.2	-0.2 to +0.1

	Q2 2002 to Q1 2003	-3.9%	-9.1 to +1.6

	Q1 2003 to Q4 2004	-0.4	-1.1 to +0.4

Suburban	Q1 1997 to Q1 2000	+0.6	-0.02 to +1.2

	Q1 2000 to Q4 2004	-0.8	-1.1 to -0.5

Rural	Q1 1997 to Q4 1999	+0.7	-0.1 to +1.5

	Q1 1999 to Q4 2004	-0.8	-1.1 to -0.5

County Poverty Level			

Low-poverty counties (<10% below FPL)	Q1 1997 to Q2 1998	+1.5	-0.2 to +3.2

	Q2 1998 to Q1 2003	-0.3	-0.6 to +0.1

	Q2 2002 to Q1 2003	-3.6	-10.2 to +3.3

	Q1 2003 to Q4 2004	-0.3	-1.2 to +0.8

Middle-poverty counties (10 to 19.9% below FPL)	Q1 1997 to Q1 1999	+0.6	+0.1 to +1.2

	Q1 1999 to Q2 2002	-0.2	-0.5 to +0.1

	Q2 2002 to Q1 2003	-3.8	-8.8 to +1.5

	Q1 2003 to Q4 2004	-0.4	-1.1 to +0.3

High-poverty counties (20+% below FPL)	Q1 1997 to Q2 1998	+1.5	-0.7 to +3.7

	Q2 1998 to Q4 2004	-0.7	-0.9 to -0.5

*In situ*			

Overall	Q1 1997 to Q2 1999	+2.5	+1.8 to +3.1

	Q2 1999 to Q4 2001	+0.4	-0.2 to +1.1

	Q4 2001 to Q4 2004	-0.6	-1.0 to -0.2

Rural/urban status			

Urban	Q1 1997 to Q2 1999	+2.3	+1.5 to +3.1

	Q2 1999 to Q4 2001	+0.4	-0.2 to +1.1

	Q4 2001 to Q4 2004	-0.7	1.1 to -0.3

Suburban	Q1 1997 to Q3 1999	+3.6	+2.0 to +5.2

	Q3 1999 to Q4 2004	-0.01	-0.4 to +0.4

Rural	Q1 1997 to Q2 2000	+2.8	+1.0 to +4.6

	Q2 2000 to Q4 2004	-0.1	-1.0 to +0.8

County poverty level			

Low-poverty counties (<10% below FPL)	Q1 1997 to Q2 1998	+3.2	+2.0% to +4.5

	Q2 1998 to Q1 2002	+0.4	-0.04 to +0.8

	Q1 2002 to Q1 2004	-1.0	-1.9 to -0.2

	Q1 2004 to Q4 2004	+5.0	-2.5 to +13.2

Middle-poverty counties (10 to 19.9% below FPL)	Q1 1997 to Q1 2000	+2.1	+1.6 to +2.7

	Q1 2000 to Q4 2004	-0.0.3	-0.6 to -0.3

High-poverty counties (20+% below FPL)	Q1 1997 to Q4 1998	+4.9	+0.9 to +9.1

	Q4 1998 to Q4 2004	-0.04	-0.6 to +0.5

CI = confidence interval; FPL = federal poverty level; Q = quarter

## Discussion

Using the largest US population-based cancer incidence resource available, we observed that substantial reductions in invasive breast cancer during the period 2001 to 2004 were more pronounced for women living in urban and low-poverty compared with other areas. Breast cancer incidence trends for rural counties, which peaked in 1999 and then declined steadily, differed from those observed in urban counties, where rates fell most dramatically after 2002. Similar but more attenuated patterns were seen for *in situ *cancers.

These data further inform the main hypotheses proposed thus far to explain recent breast cancer trends. Proportionately larger incidence drops among women living in urban and suburban than rural counties are consistent with changing patterns of HT prevalence and cessation or non-initiation but cannot rule out a possible influence of mammography saturation in rural areas. Similar to prior reports [1,2,5-7], urban and suburban trends for invasive breast cancer track temporally to nationwide HT prescription patterns, which increased until 1999, peaking at 92 million prescriptions/year, then plateaued after the release of null results regarding cardioprotection from the Heart Estrogen/Progestin Replacement Study (HERS) and unfavorable preliminary findings from the WHI [10,32-34]. Prevalence of HT plummeted by 37% to 72%, immediately following the highly publicized July 2002 termination of the WHI estrogen/progestin arm in which the experimental group was found to have higher risk of breast cancer than controls [10,11,13,14,32,35]. Haas *et al*. reported that the number of newspaper articles about the harmful effects of HT found in the WHI trial correlated with urban residence and likelihood of HT cessation/non-initiation; women in urban areas (for example, San Francisco/Bay Area) were potentially exposed to more newspaper articles and had a larger decline in the prevalence of HT use than women in more rural areas (for example, Vermont and New Mexico) [36]. Additionally, among white California Health Interview Survey (CHIS) respondents aged 50 to 74, current estrogen/progestin HT use dropped more than twice as much in urban versus rural counties between 2001 and 2003 (urban: 9.1% decline from 26.5% in 2001 to 17.4% in 2003; rural: 4.4% decline from 22.1% in 2001 to 17.7% in 2003) [37]. Because current or recent HT use appears to increase risk of invasive but not *in situ *breast cancers [38,39], the impact of widespread HT discontinuation/non-initiation should be more evident for invasive than *in situ *tumors, as observed in urban and suburban counties. Thus, our assessment of annual and quarterly trends demonstrates that the sharpest declines in invasive tumor rates among populations with the largest proportions of former HT users coincided specifically with the WHI announcement in 2002 and provides additional evidence of the correlation between population HT patterns and population breast cancer patterns.

Saturation of or decreases in mammographic screening would be expected to influence incidence trends for *in situ *tumors, which are not palpable and therefore, must be detected radiographically. In California, reductions in the proportions of women reporting biannual mammograms between 2001 and 2003 were apparent and slight in both urban and rural counties (urban: -1.4%, 86.1% in 2001 to 84.7% in 2003; rural: -0.6%, 84.1% in 2001 to 83.5% in 2003) [37]. These changes do not seem large enough to explain entirely the breast cancer drops and did not occur selectively among former HT users, which hypothetically could cause disproportionately large incidence drops [40]. More sustained declines in mammography could have impacted the rates among women living in rural counties, especially in light of their lower levels of HT utilization and the disparate drops in invasive relative to *in situ *breast cancer incidence observed here for rural versus urban/suburban populations.

We did not observe major differences in invasive breast cancer incidence trends by poverty level, perhaps because the county-level measure did not differentiate breast cancer rates well among categories. Smaller area-level measures (for example, census tract) could not be used, since NAACCR's aggregated, multi-registry data file does not include incidence data for such geographic regions. Some authors have reported more modest socioeconomic gradients in breast cancer incidence among white than non-white women [41]. Several studies have documented significant associations between HT use and individual-level markers of socioeconomic status such as education or personal income [22,42,43], but it remains unclear whether post-WHI HT utilization declined uniformly across all poverty levels. At least one study found equivalent drops [22], whereas California women with incomes 300% or more above the FPL reported considerably larger decreases in estrogen/progestin HT use (-9.8% decline from 29.6% in 2001 to 19.8% in 2003) than women below the FPL, among whom rates were stable (0.5% increase from 11.3% in 2001 to 11.8% in 2003) [37]. Regardless, the absolute number of women who stopped or did not initiate HT after 2002 was more substantial for low- versus high-poverty populations, a fact which is likely related to the marked drops in breast cancer rates in those areas. National mammographic screening patterns among white women aged 50 to 64 years were similar for all poverty levels with the percentage of screened women plateauing between 1998 and 1999 then declining after 2000 [16,19,37].

Other hypotheses set forth to explain breast cancer declines are incompatible with the trends reported in this analysis. Incidence reductions could have resulted from increases in the use of chemopreventive or other pharmaceuticals (for example, tamoxifen, raloxifene, nonsteroidal anti-inflammatory medications, and statins) thought to protect against breast cancer [1,19,44] or from changes in relevant lifestyle risk factors such as the percentage of overweight and obese individuals, number of alcoholic beverages consumed, or level of physical activity [45]. Improved detection of *in situ *tumors over the last two decades could also cause decreases in invasive cancers, if *in situ *lesions are precursors of invasive disease [44,46]. Nevertheless, because these processes are continuous and long-term [19,44] they are unlikely to account for the rapid incidence drops seen over a two-year period in the early 2000s.

Although this analysis employed data from the largest population-based cancer incidence resource in the US, including up to 60% more of the country's population than prior reports and allowing for evaluation of robust trends by annual quarter of diagnosis among multiple demographic groups, it has several important limitations. First, cancer registries generally do not collect individual-level socioeconomic information, which would supplement area-level data to help interpret trends by rural/urban and poverty status. Prior studies suggest that area-level patterns are generally consistent with but underestimate individual-level trends and more accurately reflect economic deprivation of a geographic region than of an individual. These two characteristics may be linked to one another, as residents of high-poverty areas are more likely to be poor or vice versa [27,28,47-50]. Area measures were only available at the county level and not for smaller, possibly more homogeneous, geographic designations. The size of counties varies considerably across the US [28]. To better understand the impact of including large counties for which area categorizations likely represented average values, we repeated analyses to exclude 21 US counties with 2000 Census populations of white women exceeding 1 million but saw no substantial change in observed trends (data not shown). Second, incidence rates were not adjusted for possible delays in reporting. However, a recent analysis by Jemal *et al*. found that such adjustments had little effect on breast cancer declines or other patterns [2]. Third, we were unable to stratify rates by gene expression-defined subtypes (for example, basal-like, luminal A) or hormone receptor status [51] because these characteristics are not available in the NAACCR database. Approximately 50 to 60% of all breast cancers are estimated to be hormone receptor-positive, and 30 to 35% receptor-negative [51]. Considering that the recent declines were most marked for hormone receptor-positive cancers, it is likely that regional differences would be greater for these subtypes. Finally, the detailed county-level HT and mammography utilization data used to help contextualize some of our findings were available for only California and not women nationwide; this information may not be representative of our entire study population, particularly with respect to rural and high-poverty groups.

## Conclusion

Our findings that US breast cancer incidence rate declines among white women aged 50 to 74 years were more pronounced for invasive than *in situ *tumors in urban and low-poverty compared with rural and high-poverty counties are consistent with prior analyses confirming heterogeneity in breast cancer trends by age, race/ethnicity, and tumor subtype [1-3,6,7]. These results further support an influence of population-level HT utilization patterns on population-level breast cancer incidence patterns. Identifying the specific subgroups most affected by the natural experiment that occurred when US women stopped HT en masse in 2002 remains important for understanding the future burden of breast cancer in populations relevant to epidemiology and health planning. Future investigations should seek to clarify further the possible impact of mammographic screening changes or other factors on recent breast cancer incidence, as this study was unable to rule them out.

## Abbreviations

CI: confidence interval; CINA: Cancer in North America; CHIS: California Health Interview Survey; ER+: estrogen receptor-positive; FPL: federal poverty level; HERS: Heart Estrogen/Progestin Replacement Study; HT: hormone therapy; ICD-O-3: *International Classification of Diseases for Oncology, 3rd Edition*; NAACCR: North American Association of Central Cancer Registries; NPCR: National Program of Cancer Registries; Q: quarter; SEER: Surveillance, Epidemiology, and End Results; US: United States; WHI: Women's Health Initiative.

## Competing interests

CAC has served as an expert witness for the plaintiffs in hormone therapy litigation. The other authors declare that they have no competing interests.

## Authors' contributions

AKH and CAC conceived of the study, performed the statistical analysis, interpreted the data, and drafted the manuscript. THMK, ETC, SLG, and HH participated in critical revisions of the manuscript. All authors read and approved the final manuscript.

## Pre-publication history

The pre-publication history for this paper can be accessed here:



## References

[B1] Ravdin PM, Cronin KA, Howlader N, Berg CD, Chlebowski RT, Feuer EJ, Edwards BK, Berry DA (2007). The decrease in breast-cancer incidence in 2003 in the United States. N Engl J Med.

[B2] Jemal A, Ward E, Thun MJ (2007). Recent trends in breast cancer incidence rates by age and tumor characteristics among U.S. women. Breast Cancer Res.

[B3] Katalinic A, Rawal R (2008). Decline in breast cancer incidence after decrease in utilisation of hormone replacement therapy. Breast Cancer Res Treat.

[B4] Robbins AS, Clarke CA (2007). Regional changes in hormone therapy use and breast cancer incidence in California from 2001 to 2004. J Clin Oncol.

[B5] Hausauer AK, Keegan TH, Chang ET, Clarke CA (2007). Recent breast cancer trends among Asian/Pacific Islander, Hispanic, and African-American women in the US: changes by tumor subtype. Breast Cancer Res.

[B6] Kerlikowske K, Miglioretti DL, Buist DS, Walker R, Carney PA (2007). Declines in invasive breast cancer and use of postmenopausal hormone therapy in a screening mammography population. J Natl Cancer Inst.

[B7] Glass AG, Lacey JV, Carreon JD, Hoover RN (2007). Breast cancer incidence, 1980–2006: combined roles of menopausal hormone therapy, screening mammography, and estrogen receptor status. J Natl Cancer Inst.

[B8] Cl CA, Glaser SL, Uratsu CS, Selby JV, Kushi LH, Herrinton LJ (2006). Recent declines in hormone therapy utilization and breast cancer incidence: clinical and population-based evidence. J Clin Oncol.

[B9] Glass A, Hoover RN (1988). Changing incidence of breast cancer. J Natl Cancer Inst.

[B10] Hersh AL, Stefanick ML, Stafford RS (2004). National use of postmenopausal hormone therapy: annual trends and response to recent evidence. JAMA.

[B11] Hillman JJ, Zuckerman IH, Lee E (2004). The impact of the Women's Health Initiative on hormone replacement therapy in a Medicaid program. J Womens Health (Larchmt).

[B12] Kelly JP, Kaufman DW, Rosenberg L, Kelley K, Cooper SG, Mitchell AA (2005). Use of postmenopausal hormone therapy since the Women's Health Initiative findings. Pharmacoepidemiol Drug Saf.

[B13] Buist DS, Newton KM, Miglioretti DL, Beverly K, Connelly MT, Andrade S, Hartsfield CL, Wei F, Chan KA, Kessler L (2004). Hormone therapy prescribing patterns in the United States. Obstet Gynecol.

[B14] Wysowski DK, Governale LA (2005). Use of menopausal hormones in the United States, 1992 through June, 2003. Pharmacoepidemiol Drug Saf.

[B15] Heiss G, Wallace R, Anderson GL, Aragaki A, Beresford SA, Brzyski R, Chlebowski RT, Gass M, LaCroix A, Manson JE, Prentice RL, Rossouw J, Stefanick ML (2008). Health risks and benefits 3 years after stopping randomized treatment with estrogen and progestin. JAMA.

[B16] (2007). Health, United States, with chartbook on trends in the health of Americans.

[B17] CHIS Public Use Files 2001, 2003, 2005.

[B18] Breen NA, Cronin KA, Meissner HI, Taplin SH, Tangka FK, Tiro JA, McNeel TS (2007). Reported drop in mammography: is this cause for concern?. Cancer.

[B19] Centers for Disease Control Behavioral Risk Factor Surveillance System. http://www.cdc.gov/BRFSS/.

[B20] Pfeiffer RM, Mitani A, Matsuno RK, Anderson WF (2008). Racial differences in breast cancer trends in the United States (2000–2004). J Natl Cancer Inst.

[B21] Clarke CA, Glaser SL (2007). Declines in breast cancer after the WHI: apparent impact of hormone therapy. Cancer Causes Control.

[B22] Wei F, Miglioretti DL, Connelly MT, Andrade SE, Newton KM, Hartsfield CL, Chan KA, Buist DS (2005). Changes in women's use of hormones after the Women's Health Initiative estrogen and progestin trial by race, education, and income. J Natl Cancer Inst Monogr.

[B23] SEER Registries – Characteristics of the SEER Population Compared with theTotal United States Population. http://seer.cancer.gov/registries/characteristics.html.

[B24] CINA Highlights of Cancer Incidence and Mortality in the United States and Canada, 2000–2004. http://www.naaccr.org/filesystem/pdf/CINA_highlights_00-04_v4.pdf.

[B25] Willett WC, Rockhill B, Hankinson SE, Hunter DJ, Colditz G, Harris JR, Lippman ME, Morrow M, Osborne CK (2004). Nongenetic factors in the causation of breast cancer. Diseases of the Breast.

[B26] Wang D, Dubois RN (2004). Cyclooxygenase-2: a potential target in breast cancer. Seminars in Oncology.

[B27] An Identifiability Assessment of CINA Deluxe with Area-based SES Measures. http://www.naaccr.org/filesystem/pdf/Report%20of%20RU%2006-01-05.pdf.

[B28] Singh GK, Miller BA, Hankey BF, Edwards BK (2003). Area socioeconomic variation in U.S. cancer incidence, mortality, stage, treatment, and survival, 1975–1999. NCI Cancer Surveillance Monograph Series, Number 4.

[B29] Ries LAG, Melbert D, Krapcho M, Mariotto A, Miller BA, Feuer EJ, Clegg L, Horner MJ, Howlader N, Eisner MP, Reichman M, Edwards BK, Eds (2007). SEER Cancer Statistics Review, 1975–2004.

[B30] Kim HJ, Fay MP, Feuer EJ, Midthune DN (2000). Permutation tests for joinpoint regression with applications to cancer rates. Stat Med.

[B31] Devesa SS, Donaldson J, Fears T (1995). Graphical presentation of trends in rates. Am J Epidemiol.

[B32] Haas JS, Kaplan CP, Gerstenberger EP, Kerlikowske K (2004). Changes in the use of postmenopausal hormone therapy after the publication of clinical trial results. Ann Intern Med.

[B33] Hulley S, Grady D, Bush T, Furberg C, Herrington D, Riggs B, Vittinghoff E (1998). Randomized trial of estrogen plus progestin for secondary prevention of coronary heart disease in postmenopausal women. Heart and Estrogen/progestin Replacement Study (HERS) Research Group. JAMA.

[B34] Fitzpatrick LA, Litin SC, Bell MR (2000). The Women's Health Initiative: a heart-to-HRT conversation. Mayo Clin Proc.

[B35] Kelly JP, Kaufman DW, Rosenberg L, Kelley K, Cooper SG, Mitchell AA (2005). Use of postmenopausal hormone therapy since the Women's Health Initiative findings. Pharmacoepidemiol Drug Saf.

[B36] Haas JS, Miglioretti DL, Geller B, Buist DS, Nelson DE, Kerlikowske K, Carney PA, Dash S, Breslau ES, Ballard-Barbash R (2007). Average household exposure to newspaper coverage about the harmful effects of hormone therapy and population-based declines in hormone therapy use. J Gen Intern Med.

[B37] CHIS Public Use Files 2001, 2003, 2005.

[B38] Gapstur SM, Morrow M, Sellers TA (1999). Hormone replacement therapy and risk of breast cancer with a favorable histology: results of the Iowa Women's Health Study. JAMA.

[B39] Rossouw JE, Anderson GL, Prentice RL, LaCroix AZ, Kooperberg C, Stefanick ML, Jackson RD, Beresford SA, Howard BV, Johnson KC, Kotchen JM, Ockene J (2002). Risks and benefits of estrogen plus progestin in healthy postmenopausal women: principal results from the Women's Health Initiative randomized controlled trial. JAMA.

[B40] Caan B, Habel L, Quesenberry C, Kushi L, Herrinton L (2008). Re: declines in invasive breast cancer and use of postmenopausal hormone therapy in a screening mammography population. J Natl Cancer Inst.

[B41] Krieger N, Chen JT, Waterman PD, Rehkopf DH, Yin R, Coull BA (2006). Race/ethnicity and changing US socioeconomic gradients in breast cancer incidence: California and Massachusetts, 1978–2002 (United States). Cancer Causes Control.

[B42] Friedman-Koss D, Crespo CJ, Bellantoni MF, Andersen RE (2002). The relationship of race/ethnicity and social class to hormone replacement therapy: results from the Third National Health and Nutrition Examination Survey 1988–1994. Menopause.

[B43] Brett KM, Madans JH (1997). Use of postmenopausal hormone replacement therapy: estimates from a nationally representative cohort study. Am J Epidemiol.

[B44] Robbins AS, Clarke CA (2007). Re: declines in invasive breast cancer and use of postmenopausal hormone therapy in a screening mammography population. J Natl Cancer Inst.

[B45] Vaidya JS (2008). Re: declines in invasive breast cancer and use of postmenopausal hormone therapy in a screening mammography population. J Natl Cancer Inst.

[B46] Cady B, Chung MA, Michaelson JS (2007). A decline in breast-cancer incidence. N Engl J Med.

[B47] Singh GK, Miller BA, Hankey BF, Feuer EJ, Pickle LW (2002). Changing area socioeconomic patterns in U.S. cancer mortality, 1950–1998: Part I – all cancers among men. J Natl Cancer Inst.

[B48] Singh GK, Miller BA, Hankey BF (2002). Changing area socioeconomic patterns in U.S. cancer mortality, 1950–1998: Part II – lung and colorectal cancers. J Natl Cancer Inst.

[B49] Krieger N, Chen JT, Waterman PD, Soobader MJ, Subramanian SV, Carson R (2002). Geocoding and monitoring of US socioeconomic inequalities in mortality and cancer incidence: does the choice of area-based measure and geographic level matter?: the Public Health Disparities Geocoding Project. Am J Epidemiol.

[B50] Krieger N, Williams DR, Moss NE (1997). Measuring social class in US public health research: concepts, methodologies, and guidelines. Annu Rev Public Health.

[B51] Carey LA, Perou CM, Livasy CA, Dressler LG, Cowan D, Conway K, Karaca G, Troester MA, Tse CK, Edmiston S, Deming SL, Geradts J, Cheang MC, Nielsen TO, Moorman PG, Earp HS, Millikan RC (2006). Race, breast cancer subtypes, and survival in the Carolina Breast Cancer Study. JAMA.

